# Female and diabetes are risk factors for alpha-fetoprotein and protein induced by vitamin K absence or antagonist-II negative in hepatocellular carcinoma

**DOI:** 10.1097/MD.0000000000040100

**Published:** 2024-10-18

**Authors:** Yanhui Shi, Hongli Yang, Xue Bai, Xiaoyan Liu, Qiang Li, Wenjun Du

**Affiliations:** a Cheeloo College of Medicine, Shandong University, Jinan, China; b Department of Gastroenterology, The First Affiliated Hospital of Shandong First Medical University & Shandong Provincial Qianfoshan Hospital, Jinan, China; c Department of Liver Diseases, Shandong Public Health Clinical Center, Shandong University, Jinan, China.

**Keywords:** alpha-fetoprotein, diabetes mellitus, hepatocellular carcinoma, protein induced by vitamin K absence or antagonist-II, sexual dimorphism, tumor properties

## Abstract

Hepatocellular carcinoma (HCC) is a common type of tumor with a high incidence. Alpha-fetoprotein (AFP) and protein induced by vitamin K absence or antagonist-II (PIVKA-II or des-gamma-carboxy prothrombin) are proven effective biomarkers for HCC. Combining them can enhance detection rates. However, when both AFP and PIVKA-II are negative, clinical diagnosis may be missed. This study aims to explore the risk factors for AFP and PIVKA-II negativity in HCC, thereby reducing missed diagnoses. A retrospective study enrolled 609 HCC patients at Shandong Public Health Clinical Center Affiliated with Shandong University from January 2010 to March 2022. Patients with negative AFP and PIVKA-II were the observed group, and others with at least 1 positive were controls. Epidemiological, clinical, laboratory, and radiological data were collected and analyzed to identify the frequency and factors influencing AFP and PIVKA-II negativity. Receiver operating characteristic (ROC) curves were used to assess the prediction model’s ability to detect negative AFP and PIVKA-II in HCC. Gender (*P* = .045, 95% confidence interval [95%CI] = 1.013–3.277), diabetes mellitus (*P* = .018, 95%CI = 1.151–4.422), tumor size (*P* = .000, 95%CI = 0.677–0.841), glutamate transpeptidase (*P* = .003, 95%CI = 0.239–0.737), total bilirubin (*P* = .001, 95%CI = 0.235–0.705), and hepatitis B virus-associated infections (*P* = .007, 95%CI = 0.077–0.661) were significantly associated with AFP and PIVKA-II negativity in HCC. The prediction model had an area under curve of 0.832 (*P* < .001, 95%CI = 0.786–0.877), with a sensitivity of 81.2% and specificity of 75.5% in all HCC patients. Female diabetic patients with levels closer to normal for glutamate transpeptidase and total bilirubin are more likely to develop AFP and PIVKA-II-negative HCC. Imaging is crucial for screening liver cancer in these patients.

## 1. Introduction

Primary liver cancer, with an estimated 906,000 new cases and 830,000 deaths in 2020, ranks as the 6th most common cancer and the third leading cause of cancer-related deaths globally among individuals with cancer.^[1]^ Hepatocellular carcinoma (HCC) represents the primary histologic type of liver cancer, particularly prevalent in regions with high HCC risk, mirroring the overall trends in primary liver cancer. Despite improvements in the 5-year relative survival rate of HCC since the mid-1970s, it still lags behind most other cancer types, as per the 2020 cancer statistics.^[2]^ While advancements in surgical and medical therapies have notably enhanced the prognosis for HCC patients, it remains a significant public health challenge worldwide.^[3]^ The substantial mortality and healthcare burden are often attributed to late-stage diagnoses in many patients, making those with extensive tumor burden less amenable to treatment. HCC typically arises against a backdrop of chronic liver disease, linked to conditions such as chronic hepatitis B virus (HBV) or hepatitis C virus infection, exposure to aflatoxin, alcoholic liver disease, obesity, and diabetes.^[4]^ Regular surveillance of individuals with these risk factors is widely acknowledged to enhance the early detection rate of HCC and facilitate more effective therapeutic interventions. Presently, routine screening for liver cancer commonly involves abdominal ultrasound and alpha-fetoprotein (AFP) level assessments. Notably, randomized controlled trials on a large scale have demonstrated that ultrasound screening can reduce mortality.^[5]^ However, ultrasound’s sensitivity in detecting early-stage HCC is limited, relying heavily on operator skill and being constrained by the examiner’s body size. Hence, there’s a necessity for a blood test with high sensitivity to complement ultrasonography.^[6]^

It is widely recognized that AFP serves as the most commonly used serum marker for the development of HCC. It has been identified as an independent risk factor associated with tumor differentiation status, tumor size, and patient survival time.^[7]^ However, AFP’s utility is limited by its inability to simultaneously achieve high sensitivity and specificity. When using a cutoff of 20 ng/mL, AFP exhibits good sensitivity but poor specificity, whereas at a cutoff of 200 ng/mL, sensitivity becomes inadequate despite high specificity.^[8,9]^ Moreover, in the early stages of HCC, detection rates can be as low as one-third, and AFP levels may remain within the normal range in some cases of advanced HCC. Additionally, AFP elevation can occur in benign liver diseases and embryonic tumors, posing challenges in early liver cancer identification. Consequently, efforts have been directed towards identifying novel serum markers for HCC diagnosis. Protein induced by vitamin K absence or antagonist-II (PIVKA-II) was found to be abundantly synthesized in HCC patients and proposed as an additional tumor marker for HCC in 1984.^[10]^ Several studies have demonstrated that PIVKA-II can reduce the diagnostic omission rate in AFP-negative HCC cases and exhibits remarkable specificity across various etiologies of chronic liver disease.^[11,12]^ A multicenter study suggested that PIVKA-II could serve as a screening or monitoring biomarker in high-risk populations for HCC, and its combination with AFP significantly enhances diagnostic performance compared to AFP alone.^[13]^ However, elevated levels of both AFP and PIVKA-II are not universally observed in all HCC cases.^[14]^ Therefore, the combined analysis of AFP and PIVKA-II cannot be relied upon for screening and diagnosing HCC in such instances, posing challenges for the timely treatment of these patients. Furthermore, the clinical and laboratory factors that may influence negative AFP and PIVKA-II measurements in HCC cases have not been thoroughly investigated.

In our study, we conducted a retrospective analysis to explore potential clinical and laboratory factors associated with negative PIVKA-II and AFP in HCC. Our aim is to broaden the screening scope for liver cancer in high-risk groups and decrease the rate of missed diagnoses to ensure timely treatment.

## 2. Materials and methods

### 2.1. Patient and sets

This cross-sectional study retrospectively enrolled 609 patients diagnosed with HCC between January 21, 2010, and March 3, 2022, at the Shandong Public Health Clinical Center Affiliated with Shandong University. These patients were categorized into 2 groups: 110 were HCC patients with negative AFP and PIVKA-II, while the remaining 499 had positive AFP or PIVKA-II. Additionally, among the 499 patients, the difference between single-marker positivity and positivity for both markers was further analyzed. Diagnosis of HCC was based on the 2021 Practice Guidance from the American Association for the Study of Liver Diseases.^[15]^ The final diagnosis of hepatocellular carcinoma was classified based on whether patients had retained liver tissue for pathology. Out of the total, 386 patients (63.4%) underwent pathological examination following hepatectomy or fine-needle aspiration for HCC diagnosis. The remaining 223 patients (36.6%) with advanced HCC, characterized by impaired liver function, portal vein invasion, or metastasis, did not undergo invasive surgery. Instead, HCC diagnosis in this group relied on various combined imaging modalities such as ultrasound, enhanced computed tomography (CT), magnetic resonance imaging, and AFP levels exceeding 400 g/L. Among these patients, 51 individuals with nodules ≥ 2 cm on ultrasound underwent dynamic contrast-enhanced CT for clinical diagnosis of HCC. The remaining 36 patients with nodules < 2 cm on ultrasound underwent both dynamic contrast-enhanced CT and multi-parametric magnetic resonance imaging, which revealed characteristic imaging features indicative of HCC for clinical diagnosis. The remaining 136 patients with elevated serum AFP levels were diagnosed with HCC based on dynamic contrast-enhanced CT. Patients initially diagnosed with hepatocellular carcinoma with comprehensive medical records were included in the study, while those with further consultations, secondary liver metastases, prolonged obstructive jaundice, taking anticoagulants such as biscoumarin, vitamin K deficiency, and biliary malignancies were excluded. The study was approved by the ethics committee of Shandong Public Health Clinical Center (SPHCC-2021-17), and informed consent was obtained from each patient or their family members. The severity of liver dysfunction was assessed using the Child-Pugh and model for end-stage liver disease scores, while HCC tumor staging was determined using the Barcelona score.

### 2.2. Data collection

Demographic data, exposure history, symptoms, signs, laboratory test results, and imaging findings were retrieved from the electronic medical record system for newly diagnosed HCC patients.

### 2.3. Statistical analysis

The collected data were entered into Microsoft Excel for Macintosh (version 16.30), and all statistical analyses were conducted using Statistical Product and Service Solutions (version 15.0). Measurement data were presented as means with 1st to 3rd interquartile ranges or as numbers with percentages. The risk factors of liver cancer, such as genetic factors, metabolic factors, immune factors, lifestyle factors, and infection factors, and the factors that may affect AFP and PIVKA-II, such as liver function, kidney function, inflammatory indicators, tumor type, and stage, were included in the univariate analysis as independent variables,^[16]^ and variable screening was performed using appropriate one-way analysis of variance for different data types. Skewed distribution data were analyzed using the Mann–Whitney *U* test, while categorical variables were assessed using Fisher exact test or Pearson *χ*^2^ test. Binary logistic regression analysis was utilized to further evaluate statistically significant variables. Tables 1 and 2 present the independent variables included in the binary logistic regression analysis. The sample size for binary logistic regression was assessed using the events per variable method to ensure appropriateness.^[17]^ Receiver operating characteristic curves were employed to assess the predictive model’s ability to detect negative AFP and PIVKA-II in HCC.

**Table 1:** Comparison demographic and tumor characteristics of patients between groups A and B.

Variables	A	B			*Z*/*x*^2^	*P*
		C	D	E		
	N = 110	N = 40	N = 114	N = 345		
Sex (female/male) n	32/78	7/33	18/96	66/279	6.588	**.010**
Age n (P25, P75)	61 (49.8,67.0)	56 (40,66)	54 (40,65)	56 (48,66)	‐0.991	.321
Etiology n (%)					8.998	**.030**
HBV	90 (81.8%)	37 (92.5%)	95 (83.3%)	319 (92.5%)		
HCV	4 (3.6%)	2 (5.0%)	5 (4.4%)	10 (2.9%)		
Alcohol liver	6 (5.5%)	0 (0.0%)	7 (6.1%)	6 (1.7%)		
Other reasons	10 (9.1%)	1 (0.3%)	7 (6.1%)	10 (2.9%)		
Family history n (%)					1.623	.444
No	86 (78.2%)	32 (80.0%)	91 (79.8%)	287 (83.2%)		
Father	10 (9.1%)	4 (10.0%)	12 (10.5%)	29 (8.4%)		
Mother	14 (12.7%)	4 (10.0%)	11 (9.6%)	29 (8.4%)		
Smoking history n (%)	31 (28.2%)	10 (25.0%)	35 (30.7%)	101 (29.3%)	0.051	.822
Complication/complication disease n (%)						
Diabetes mellitus	25 (22.7%)	4 (10.0%)	14 (12.3%)	61 (12.2%)	8.198	**.004**
Cirrhosis	103 (93.6%)	35 (87.5%)	104 (91.2%)	334 (96.8%)	0.332	.565
Fatty liver	9 (8.2%)	1 (2.5%)	4 (3.5%)	21 (4.2%)	3.083	.081
Another clinical character						
Tumor size n (P25,P75)	2.0 (1.3,3.7)	2.0 (1.3,2.5)	4.8 (2.7,8.2)	7.0 (3.8,11.3)	8106.0	**.000**
Tumor type n(%)					49.561	**.000**
Nodular type	92 (83.6%)	31 (77.5%)	53 (46.5%)	149 (43.2%)		
Diffuse type	4 (3.6%)	2 (5.0%)	10 (8.8%)	33 (9.6%)		
Massive type	14 (12.7%)	7 (17.5%)	51 (44.7%)	163 (47.2%)		
Tumor number n (%)					29.487	**.000**
1	76 (69.1%)	28 (70.0%)	62 (54.4%)	120 (34.8%)		
2	7 (6.4%)	3 (7.5%)	9 (7.9%)	13 (3.8%)		
3	1 (0.9%)	2 (5.0%)	3 (2.6%)	4 (1.2%)		
≥4	26 (23.6%)	7 (17.5%)	40 (35.1%)	208 (62.3%)		
Neoplasm metastasis n (%)	11 (10.0%)	1 (2.5%)	27 (23.7%)	85 (24.6%)	8.888	**.003**
MELD grade n (%)					5.608	.132
<15	97 (88.2%)	37 (92.5%)	98 (86.0%)	259 (75.1%)		
15–19	6 (5.5%)	1 (2.5%)	6 (5.3%)	35 (10.1%)		
20–29	3 (2.7%)	1 (2.5%)	7 (6.1%)	31 (9.0%)		
≥30	4 (3.6%)	1 (2.5%)	3 (2.6%)	20 (5.8%)		
Child-pugh score n (%)					24.508	**.000**
A	61 (55.5%)	21 (52.5%)	45 (39.5%)	87 (25.2%)		
B	28 (25.5%)	11 (27.5%)	44 (38.6%)	129 (37.4%)		
C	21 (19.1%)	8 (20.0%)	25 (21.9%)	129 (37.4%)		
Barcelona clinic liver cancer score n (%)					40.083	**.000**
A	67 (60.9%)	24 (60.0%)	44 (38.6%)	78 (22.6%)		
B	11 (10.0%)	5 (12.5%)	22 (19.3%)	64 (18.6%)		
C	10 (9.1%)	3 (7.5%)	23 (20.2%)	74 (21.4%)		
D	22 (20.0%)	8 (20.0%)	25 (21.9%)	129 (37.4%)		

Group A, AFP < 25 and PIVAK-II < 40; group B, AFP ≥ 25 or PIVAK-II ≥ 40; group C, AFP ≥ 25 and PIVAK-II < 40; group D, AFP < 25 and PIVAK-II ≥ 40; group E, AFP ≥ 25 and PIVAK-II ≥ 40; family history, family history of liver cancer. The bold values indicate a statistically significant *P*-value for their analysis of that variable (*P* < .05).

HBV = hepatitis B virus; HCV = hepatitis C virus; MELD = model for end-stage liver disease.

**Table 2:** Laboratory test index between the groups A and B.

	Normal range	A	B			*Z*/*x*^2^	*P*
			C	D	E		
		N = 110	N = 40	N = 114	N = 345		
ALT (U/L)	0–40					11.307	**.001**
<40 U/L		65 (59.1%)	21 (52.5%)	60 (52.6%)	126 (36.5%)		
≥40 U/L		45 (40.9%)	19 (47.5%)	54 (47.4%)	219 (63.5%)		
AST (U/L)	0–40					42.262	**.000**
<40 U/L		57 (51.8%)	17 (42.5%)	48 (42.1%)	42 (12.2%)		
≥40 U/L		53 (48.2%)	23 (57.5%)	66 (57.9%)	303 (87.8%)		
TB (µmol/L)	0–17.1					31.292	**.000**
<17.1		45 (40.9%)	12 (30.0%)	25 (21.9%)	47 (13.6%)		
≥17.1		65 (59.1%)	28 (70.0%)	89 (78.1%)	298 (86.4%)		
DB (µmol/L)	0–6.8					28.601	**.000**
<6.8		45 (40.9%)	14 (35.0%)	29 (25.4%)	45 (13.0%)		
≥6.8		65 (59.1%)	26 (65.0%)	85 (78.1%)	300 (87.0%)		
ALB (g/L)	35–55					9.099	**.003**
<35		28 (25.5%)	10 (25.0%)	25 (21.9%)	34 (9.9%)		
≥35		82 (74.5%)	30 (75.0%)	89 (78.1%)	311 (90.1%)		
TG (mmol/L)	0.56–1.70					0.702	.402
0.56–1.7		92 (83.6%)	31 (77.5%)	86 (75.4%)	283 (82.0%)		
Other numerical		18 (16.4%)	9 (22.5%)	28 (24.6%)	62 (18.0%)		
CH(mmoL/ L)	2.60–6.19					0.712	.399
2.60–6. 19		91 (82.7%)	35 (87.5%)	88 (77.2%)	272 (78.8%)		
Other numerical		19 (17.3%)	5 (12.5%)	26 (22.8%)	73 (21.2%)		
ALP (U/L)	40–150					60.767	**.000**
40–150		92 (83.6%)	36 (90.0%)	70 (61.4%)	180 (52.2%)		
Other numerical		18 (16.4%)	4 (10.0%)	44 (38.6%)	165 (47.8%)		
GGT (U/L)	12–64					60.767	**.000**
12–64		72 (65.5%)	26 (65.0%)	37 (37.5%)	70 (20.3%)		
Other numerical		38 (34.5%)	14 (35.0%)	77 (67.5%)	275 (79.7%)		
LDH (U/L)	120–150					12.188	**.000**
120–250		58 (52.7%)	24 (60.0%)	48 (42.1%)	102 (29.6%)		
Other numerical		52 (47.3%)	16 (40.0%)	66 (57.9%)	243 (70.4%)		
BUN (mmol/L)	3.2–7.1					7.355	**.007**
3.2–7. 1		94 (85.5%)	37 (92.5%)	85 (74.6%)	243 (70.4%)		
Other numerical		16 (14.5%)	3 (7.5%)	29 (25.4%)	102 (29.6%)		
Cr (µmol/L)		53–106 (male); 44–97 (female)				2.222	.136
53–106 (M); 44–97 (F)		88 (80.0%)	34 (85.0%)	84 (73.7%)	247 (71.6%)		
Other numerical		22 (20.0%)	6 (15.0%)	30 (26.3%)	98 (28.4%)		
HGB (g/L)	120–160					1.251	.263
120–160		64 (58.2%)	32 (80.0%)	58 (50.9%)	171 (49.6%)		
Other numerical		46 (41.8%)	8 (20.0%)	56 (49.1%)	174 (50.4%)		
INR	0.8–1.2					10.991	**.001**
0.8–1.2		44 (40.0%)	17 (19.1%)	39 (34.2%)	66 (42.5%)		
Other numerical		66 (60.0%)	23 (57.5%)	75 (65.8%)	279 (80.9%)		
PT(s)	8.8–13.8					4.276	**.039**
8.8–13.8		55 (50.0%)	20 (50.0%)	54 (47.4%)	122 (35.3%)		
Other numerical		55 (50.0%)	20 (50.0%)	60 (52.6%)	223 (64.6%)		
PTA (%)	70–130					7.676	**.006**
70–130		75 (68.2%)	28 (70.0%)	70 (61.4%)	170 (49.3%)		
Other numerical		35 (31.8%)	12 (30.0%)	44 (38.6%)	175 (50.7%)		
DR (µg/mL)	0–0.5					22.557	**.000**
<0.5		64 (58.2%)	25 (62.5%)	49 (43.0%)	95 (27.5%)		
≥0.5		46 (41.8%)	15 (37.5%)	65 (57.0%)	250 (72.5%)		
CEA (g/L)	0–5					0.989	.320
<5		70 (63.6%)	30 (75.0%)	84 (73.7%)	228 (66.1%)		
≥5		40 (36.4%)	10 (25.0%)	30 (26.3%)	117 (33.9%)		
CA199 (kU/L)	0–40					0.257	.612
<40		74 (67.3%)	32 (80.0%)	82 (71.9%)	209 (60.6%)		
≥40		36 (32.7%)	8 (20.0%)	32 (28.1%)	136 (39.4%)		
LgG (g/L)	7–16					3.032	.220
7–16		38 (34.5%)	9 (22.5%)	42 (36.8%)	107 (31.0%)		
Other numerical		72 (65.5%)	31 (77.5%)	72 (63.2%)	238 (69.0%)		
SAA ELISA (mg/L)	0–10					2.488	.115
<10		23 (20.9%)	11 (27.5%)	13 (11.4%)	50 (14.5%)		
≥10		87 (79.1%)	29 (72.5%)	101 (88.6%)	295 (85.5%)		
PCT (mU/L)	2–10					15.000	**.001**
2–10		17 (15.5%)	6 (15.0%)	13 (11.4%)	12 (3.5%)		
Other numerical		93 (84.5%)	34 (85.0%)	101 (88.6%)	333 (96.5%)		
CPR (mg/L)	0.068–8.2					5.608	.132
0.068–8.2		56 (50.9%)	27 (67.5%)	42 (36.8%)	83 (24.1%)		
Other numerical		54 (49.1%)	13 (32.5%)	72 (63.2%)	262 (75.9%)		

Group A, AFP < 25 and PIVAK-II < 40; group B, AFP ≥ 25 or PIVAK-II ≥ 40; group C, AFP > 25 and PIVAK-II < 40; group D, AFP < 25 and P-IVAK-II ≥ 40; group E, AFP ≥ 25 and PIVAK-II ≥ 40.

The bold values indicate a statistically significant *P*-value for their analysis of that variable (*P* < .05).

ALB = albumin, ALP = alkaline phosphatase, ALT = alanine aminotransferase, AST = aspartate aminotransferase, BUN = blood urea nitrogen, CA199 = carbohydrate antigen 199, CEA = carcinoembryonic antigen, CH = cholesterol, Cr = creatinine, CRP = C-reactive protein, DB = direct bilirubin, DR = D-dimer, GGT = γ-glutamyl transpeptidase, HGB = hemoglobin, IgG = immunoglobin G, INR = international normalized ratio, LDH = lactate dehydrogenase, PCT = procalcitonin, PT = prothrombin, PTA = prothrombin activity, SAA ELISA = serum amyloid A, TB = total bilirubin, TG = triglyceride.

## 3. Result

### 3.1. Baseline patient characteristics

**Figure 1.**
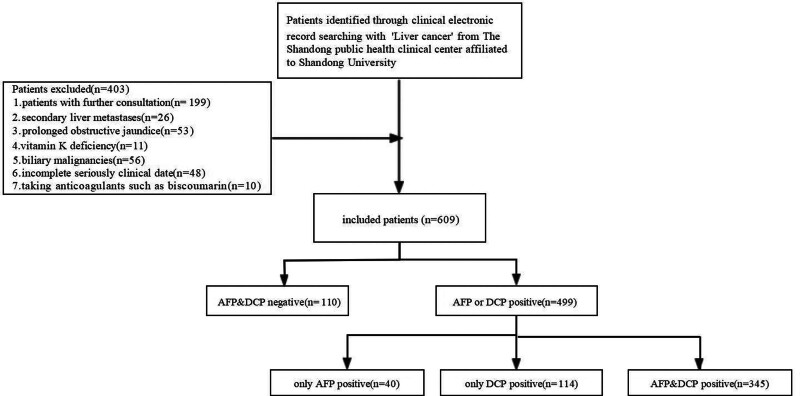
The flowchart of patient selection. Patients identified through clinical electronic record searching with “Liver cancer” from The Shandong public health clinical center affiliated to Shandong University. Patients with further consultations, secondary liver metastases, prolonged obstructive jaundice, taking anticoagulants such as biscoumarin, vitamin K deficiency, and biliary malignancies were excluded, 609 patients were enrolled in the study.

The patient inclusion and exclusion process for this study is depicted in Figure 1. A total of 609 patients diagnosed with hepatocellular carcinoma were analyzed, divided into the experimental group A (n = 110) and the control group B (n = 499). Further screening from the 499 patients categorized into single tumor marker-positive patients yielded 3 groups: C, D, and E. Tables 1 and 2 present the demographic, clinical, and cancer characteristics of patients across the 5 groups. Notably, HBV infection remains the most common etiology of HCC in China.

### 3.2. Influence factors for AFP and PIVKA-II of HCC

Chi-squared test analysis revealed several factors significantly associated with negative AFP and PIVKA-II in liver cancer within this population (Table 1). These factors included higher HCC-nonB ratios, female gender, diabetes, smaller or fewer tumors, nodular hepatocellular carcinoma, lower metastasis rates, and levels closer to normal for various biochemical markers such as aspartate transaminase, alanine aminotransferase transaminase, albumin, total bilirubin (TB), direct bilirubin, alkaline phosphatase, glutamyl transpeptidase (GTT), lactate dehydrogenase, blood urea nitrogen, prothrombin time, activated partial thromboplastin time, international normalized ratio, D-dimer, procalcitonin, and C-reactive protein. Additionally, lower Barcelona Clinic Liver Cancer and Child Turcotte Pugh classification were associated with negative AFP and PIVKA-II (*P* < .05 for each).

In multivariate analyses (Table 3), female gender, diabetes, higher HCC-nonB ratios, smaller tumors, and levels closer to normal for glutamate transpeptidase and total bilirubin remained significantly associated with negative AFP and PIVKA-II in liver cancer (*P* < .05 for each).

**Table 3:** Binary logistic regression analysis to identify influencing factors for AFP and PIVKA-II negative HCC.

	B	*P*-value	OR	95%CI	
				Lower	Upper
Sex (male/female)	0.600	**.045**	1.822	1.013	3.277
Etiology		**.024**			
HBV	‐1.489	**.007**	0.226	0.077	0.661
HCV	‐1.601	.050	0.202	0.041	1.002
Alcohol liver	‐0.519	.530	0.595	0.118	3.001
Diabetes	0.814	**.018**	2.256	1.151	4.422
Tumor size	‐0.282	**.000**	0.754	0.677	0.841
GGT	‐0.868	**.003**	0.420	0.239	0.737
TB	‐0.898	**.001**	0.407	0.235	0.705
Constant quantity	1.989	**.002**	7.305		

Use other reasons as a control group of etiology. HBV, HCV, and alcoholic liver were compared with other causes, respectively. The bold values indicate a statistically significant *P*-value for their analysis of that variable (*P* < .05).

95%CI = confidence interval; B = beta regression coefficient, GGT = glutamyl transpeptidase; HBV = hepatitis B virus; HCV = hepatitis C virus; OR = odds ratio; TB = total bilirubin.

**Figure 2.**
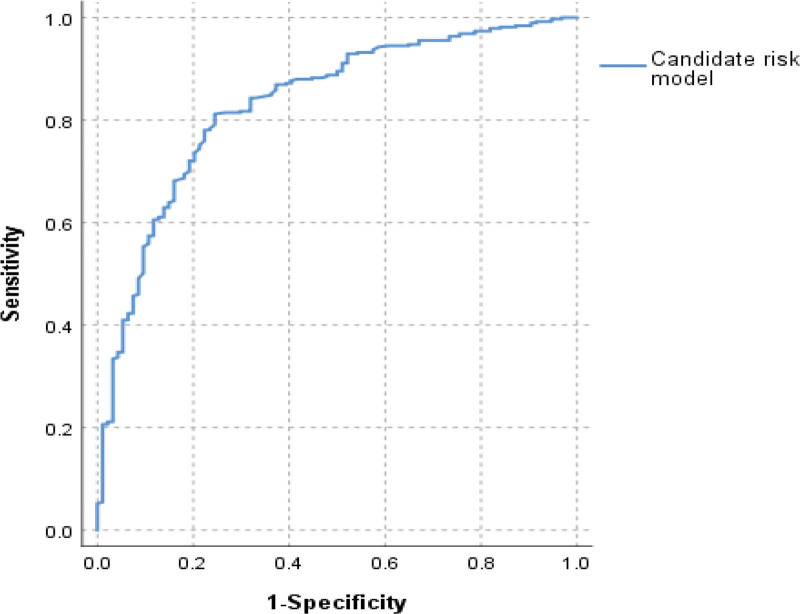
Receiver operating characteristic curves of candidate risk model. (A) ROC curves were used to evaluate the predictive model’s ability to detect negative AFP and PIVKA-II in HCC. ROC curve = receiver operating characteristic curve.

Using a binary logistic regression model, the combined area under curve for 6 significantly correlated indicators was 0.832 for diagnosing negative AFP and PIVKA-II in all 609 HCC patients (Table 4 and Fig. 2).

**Table 4:** Receiver operating characteristic analysis for the candidate risk model in all selected patients.

Indicators	AUC	SE	*P*-value	95%CI	
				Lower	Upper
Candidate risk model	0.832	0.023	.000	0.786	0.877

95%CI = 95% confidence interval, AUC = area under curve, SE = standard error.

## 4. Discussion

HCC poses significant medical and economic burdens due to its high mortality and morbidity rates. Early screening and diagnosis of liver cancer are crucial for effective treatment. The undeniable fact remains that combining AFP and PIVKA-II improves diagnosis and recurrence rates in HCC patients. In our study, we aimed to identify factors influencing negative AFP and PIVKA-II indicators to offer screening recommendations for high-risk patients who may be missed. We found that gender, as an uncontrollable factor, closely correlated with negative AFP and PIVKA-II occurrence in HCC. Additionally, diabetes, small tumor size, non-HBV infection-related HCC, reduced TB, and glutamate transpeptidase (GGT) levels also influenced the AFP and PIVKA-II positivity rates in HCC patients.

Sexual dimorphism in liver cancers was initially observed in mice in the late 1930s, with female mice showing resistance to liver cancer.^[18]^ This dimorphism is also evident in human liver diseases, as epidemiological studies indicate that the incidence of liver cancer in males is 2 to 4 times higher than in females. The liver serves as a target organ for sex hormones, suggesting that differences in sex hormone levels and sex hormone-specific gene expression play a significant role in this sexual dimorphism.^[19]^ Chronic HBV infection is the primary cause of liver cancer development globally. Studies have reported higher androgen levels and more active androgen receptor alleles in male HBV carriers, particularly in high-risk populations for HCC.^[20]^ Androgens enhance HBV gene replication and transcription by binding directly to the androgen response element in the enhancer I of the HBV genome, resulting in a synergistic oncogenic effect with HBV. However, this effect is not observed in females.^[20–22]^ Additionally, the androgen receptor can inhibit the expression of interleukin-12 A by directly binding to the interleukin-12 A promoter region, thereby suppressing natural killer cell cytotoxicity against liver cancer cells. The AR can also collaborate with cell cycle-related kinases to activate oncogenic β-catenin in hepatocytes, promoting hepatocyte carcinogenesis.^[19]^ In contrast, estrogen, acting through estrogen receptor alpha, a tumor suppressor protein, may exert a protective role. Estrogen receptor alpha expression is negatively correlated with HBV replication, tumor size, and tumor stage.^[23]^ Estrogen has been shown to inhibit the development of HBV-related HCC by reducing HBV RNA transcription. Additionally, estrogens can inhibit interleukin-6 by reducing activation via Myd88-induced nuclear factor kappa-B, thereby suppressing the progression of liver fibrosis and chronic liver disease.^[19,24,25]^ Our study identified sex as an independent risk factor for missed diagnosis when screening for liver cancer using AFP and PIVKA-II. Women were more likely to produce negative results, potentially due to the effects of sex hormones mentioned above.

The relationship between HBV and HCC is well-established, with hepatitis B-associated HCC exhibiting higher levels of AFP compared to other etiologies.^[26]^ Zhang et al concluded that the hepatitis B virus X protein triggers AFP transcription, leading to increased AFP expression in HBV-associated HCC.^[27]^ In our study, we observed that HBV-related HCC patients are more likely to have elevated AFP and PIVKA-II levels than those with nonviral and non-alcohol-related HCC. Interestingly, Ricco et al reported that PIVKA-II levels in patients with other types of HCC are significantly higher than in those with viral HCC at the initial visit. These differences could be attributed to variations in health examinations among patient groups in different countries. Furthermore, higher PIVKA-II levels in nonviral HCC patients may result from more rigorous ultrasound surveillance, leading to the detection of more advanced disease at the time of initial diagnosis.^[28]^ Previous studies have indicated a positive correlation between both AFP and PIVKA-II levels and tumor size.^[29–31]^ Consistent with these findings, our study identified tumor size as an independent factor associated with abnormally elevated levels of PIVKA-II and AFP in HCC patients. Additionally, certain tumor properties such as tumor number, type, and metastasis were associated with abnormal elevation of PIVKA-II and AFP levels, although this association was only significant in univariate analysis. Notably, tumor size exerted a greater influence on PIVKA-II and AFP levels than tumor number, type, or metastasis, suggesting that patients with positive AFP and PIVKA-II may have a poorer prognosis. We also observed that PIVKA-II-negative patients tended to have smaller tumor diameters compared to AFP-negative HCC patients, consistent with the findings of Nakamura et al.^[32]^ This suggests that PIVKA-II may be more useful for diagnosing HCC in cases of large tumors compared to AFP, but less effective for small tumors.

As a crucial metabolic organ, the liver plays a pivotal role in regulating blood glucose levels. Numerous epidemiological studies have established an association between type 2 diabetes mellitus (T2DM) and the incidence of HCC.^[33,34]^ Miele et al observed a significant increase in HCC risk among patients with longer durations of T2DM.^[35]^ Additionally, Shaoling et al found in a retrospective study that T2DM is linked to lower AFP levels and a higher risk of high-grade HCC. While previous studies have explored the relationship between T2DM and liver cancer risk,^[36]^ our study also revealed that HCC patients with diabetes mellitus were more likely to exhibit negative AFP and abnormal prothrombin events. This suggests that diabetes mellitus may lead to reduced serum AFP and PIVKA-II levels, potentially increasing the false-negative rate of AFP and PIVKA-II screening for HCC in T2DM patients. Clinicians should be mindful of this phenomenon, indicating that the current AFP and PIVKA-II thresholds may not be suitable for early HCC screening in diabetic populations.

Prior research has highlighted that PIVKA-II levels and AFP can increase to varying degrees due to liver regeneration induced by necrosis and inflammation during liver injury, with PIVKA-II being more specific than AFP in liver cancer diagnosis.^[37]^ However, less attention has been paid to the confounding factors of liver injury and liver function affecting PIVKA-II and AFP levels. A cross-sectional study by Hana et al found a positive association between the 2 markers and total bilirubin levels in HCC patients.^[26]^ Furthermore, our research revealed a significant association between abnormal PIVKA-II and AFP levels and TB and GGT in HCC patients. Unfortunately, the underlying mechanisms linking TB and GGT to increased PIVKA-II and AFP levels in HCC patients remain unclear. We speculate that elevated TB and GGT are common in various liver disorders, particularly those involving liver injury with cholestasis. Previous studies have suggested that vitamin K deficiency, a typical complication in patients with chronic cholestasis, may also impact PIVKA-II and AFP levels, as vitamin levels were negatively correlated with serum TB levels.^[31]^ This suggests that levels closer to normal for GGT and TB in hepatopath may influence the diagnostic efficacy of PIVKA-II and AFP for liver cancer.

Several limitations are present in the current study. Firstly, this study is a retrospective analysis with limited sample size obtained from patient data, which may be influenced by subjective factors due to the acquisition history with patients or physicians, and there was no follow-up conducted to explore differences in outcomes among different groups of patients. Additionally, the data is derived from a single hospital in China, which is limited by the specific geographical, cultural and medical resource distribution, and the incidence, etiology, treatment level and survival status of liver cancer patients in different regions may be significantly different. For instance, hepatitis-related backgrounds are particularly common among liver cancer patients in Asia while alcohol consumption remains a major cause of liver cancer in Western countries. Developed countries may place a greater emphasis on the prevention and treatment of liver disease, leading to variations in disease severity and survival rates among patients with liver cancer based on different economic levels. These variations may lead to an overrepresentation of HBV-infected liver cancer cases and a smaller proportion of patients with mild disease within our study population, thereby limiting the generalizability and widespread applicability of the research findings. Although efforts were made to exclude potential confounders in the exclusion criteria, some biases may still exist. For example, all patients with long-term obstructive jaundice were excluded from this study, and it is possible that some patients with mild disease without vitamin K deficiency or infection were also excluded from this study. Finally, the study included HCC patients with various etiologies, precluding a detailed evaluation of the effects of these factors on PIVKA-II and AFP. In summary, the impact of these factors on the diagnostic efficacy of the 2 tumor markers should be confirmed through prospective studies with larger sample sizes.

## Author contributions

**Data curation:** Yanhui Shi, Hongli Yang, Xue Bai, Xiaoyan Liu, Qiang Li.

**Funding acquisition:** Qiang Li.

**Writing – original draft:** Yanhui Shi.

**Writing – review & editing:** Wenjun Du.
